# Supplementary material for: Assessing the real-world performance of xylazine test strips for community-based drug checking in Los Angeles
Source: Harm Reduct J. 2026 Feb 8;23:53. doi: 10.1186/s12954-026-01396-z (PMC12977899; doi:10.1186/s12954-026-01396-z)

**Supplemental Methods**

**Test Strip Procedure**

Samples were prepped in the field using a 5-10 mg microscoop to place 1 mg of sample into a 1.5 mL graduated Eppendorf vial. Drug checking technicians were trained to identify what 1 mg of sample in the tube would look like, using an image of an Eppendorf tube with 1 mg of sugar weighed on a scale. After 1 mg of sample were added to the Eppendorf vial, 1 mL of sterile water was added from a disposable water ampoule, using the measurements on the side of the vial. The vial was capped, and technicians manually agitated the vial for 10 seconds to ensure the contents of the vial were adequately mixed. A single test strip was dipped into the vial, inserting the wavy liquid icon below the max line of the test strip until the technician saw water begin to run up the test strip, about 15 seconds. Once water ran up the strip, the test strip was removed from the vial and placed on a flat, nonabsorbent surface. After 2 minutes, test strip results were read. Senior technicians oversaw all drug checking procedures, to ensure that the appropriate procedures were followed.

**Steps from training manual for XTS strips:**

1. 1 mg of the same sample and 1.0 ml of filtered water were added to an Eppendorf vial. The vial was agitated manually for ten seconds.
2. We then employed xylazine test strips from Wisebatch to check each sample for the presence of xylazine. The wavy side of each strip was inserted into the dissolved solution until water began to run up the strip, about 15 seconds. Technicians are trained to dip the strip taking care to not insert it past the thick blue max line. After water began to run up the XTS, the strip was placed flat on a clean, non-absorbent surface to allow the solution to run up the strip to run via capillary action.
3. After 2 minutes, two trained technicians read the test strip results. In the rare case of disagreement between technicians, or an inconclusive or invalid result, a second strip was employed, providing a definitive result in all cases.
4. Interpretation of test strip results: One line is positive, two lines are negative, and the test is invalid if no lines show or there is no control line.

**Sample Disposal Procedure**

To dispose of the samples, we used nontoxic DisposeRx powder to render the drug samples inert in a viscous gel.

1. The sample powder, sample solution in the Eppendorf vial, a DisposeRx packet, and 15 mL of filtered water were added to a 22 mL vial at the end of each drug checking clinic.
2. The vial was then agitated manually until the gel formed.
3. This vial was then safely discarded into the trash.

**Laboratory Sample Preparation**

1. To collect samples for lab-based testing, drug checking technicians used a 5mg microscoop to place 1 scoop of sample in a labelled 2mL amber glass vial containing about 0.5mL of acetonitrile.
2. The vial is then tightly capped and sealed with a strip of parafilm to prevent leakage.
3. This vial is then placed in the corresponding labelled plastic envelope.
4. All sample envelopes are then placed in a clear zippered bag with a sorbent material, to be submitted to NIST for qualitative analysis with DART-MS and quantitative analysis with LC-MS/MS.

**Supplemental Results**

| **Testing Results** | | | | |
| --- | --- | --- | --- | --- |
|  |  | **Mass Spectrometry** | |  |
|  |  | ***Negative*** | ***Positive*** |  |
| **Test Strip** | ***Negative*** | 614 | 31 | 645 |
|  | ***Positive*** | 36 | 34 | 70 |
|  |  | 650 | 65 | 715 |
|  | **Summary Statistics** | | | |
|  | Sensitivity | 52.31% | PPV | 48.57% |
|  | Specificity | 94.46% | NPV | 95.19% |
|  |  |  |  |  |

**Supplemental Table 1. Predictive Performance of Xylazine Test Strips Among Full Sample, Not limited to Fentanyl Positive Samples**

A comparison is shown between results from xylazine test strips to confirmatory testing based on DART-MS. Sensitivity, specificity, positive predictive value (PPV), and negative predictive value (NPV) are also calculated. Row and column marginal sums are also shown.

| **#** | **Drug Contents on DART-MS** | **Lidocaine** | **Ketamine** |
| --- | --- | --- | --- |
| 1 | Cocaine, Lidocaine, Ketamine, Benzoylecgonine, Acetaminophen | 1 | 1 |
| 2 | 4-ANPP, Acetaminophen, Caffeine, Cocaine, Fentanyl, Mannitol, Norfentanyl, Theobromine, Lidocaine, Aniline | 1 | 0 |
| 3 | Acetaminophen, Cocaine, Fentanyl, Lidocaine, Quetiapine, Caffeine | 1 | 0 |
| 4 | 4-ANPP, Acetaminophen, Fentanyl, Lidocaine, Fluorofentanyl | 1 | 0 |
| 5 | 4-ANPP, Acetaminophen, Fentanyl, Lidocaine | 1 | 0 |
| 6 | 4-ANPP, Acetaminophen, Caffeine, Fentanyl, Lidocaine, Norfentanyl, Methamphetamine, Aniline | 1 | 0 |
| 7 | Acetaminophen, Fentanyl, Lidocaine, 4-Anpp, Phenethyl 4-Anpp | 1 | 0 |
| 8 | 4-ANPP, Acetaminophen, Bis(2,2,6,6-Tetramethyl-4-Piperidyl) Sebacate, Fentanyl, Lidocaine, Methamphetamine , Mannitol, Phenethyl 4-Anpp | 1 | 0 |
| 9 | 4-ANPP, Acetaminophen, Bis(2,2,6,6-Tetramethyl-4-Piperidyl) Sebacate, Fentanyl, Lidocaine, Mannitol, Aniline, Fluorofentanyl | 1 | 0 |
| 10 | 4-ANPP, Acetaminophen, Bis(2,2,6,6-Tetramethyl-4-Piperidyl) Sebacate, Fentanyl, Lidocaine, Mannitol, Aniline, Fluorofentanyl, Ethyl 4-Anpp | 1 | 0 |
| 11 | 4-ANPP, Acetaminophen, Fentanyl, Fluorofentanyl, Lidocaine | 1 | 0 |
| 12 | Fentanyl, Lidocaine, Mannitol, 4-ANPP, Phenethyl 4-ANPP | 1 | 0 |
| 13 | Acetaminophen, Fentanyl, Lidocaine | 1 | 0 |
| s | 4-ANPP, Bis(2,2,6,6-Tetramethyl-4-Piperidyl) Sebacate, Fentanyl, Lidocaine, Phenacetin | 1 | 0 |
| 15 | Bis(2,2,6,6-Tetramethyl-4-Piperidyl) Sebacate, Caffeine, Lidocaine, Mannitol, Fentanyl | 1 | 0 |
| 16 | 4-ANPP, Acetaminophen, Fentanyl, Lidocaine, Ethyl 4-ANPP | 1 | 0 |
| 17 | Acetaminophen, Fentanyl, Lidocaine, Mannitol, NPP | 1 | 0 |
| 18 | Acetaminophen, Fentanyl, Lidocaine, Fluorofentanyl, 4-ANPP | 1 | 0 |
| 19 | 4-ANPP, Bis(2,2,6,6-Tetramethyl-4-Piperidyl) Sebacate, Fentanyl, Lidocaine, 1-Phenethylpiperidin-4-ol, Mannitol | 1 | 0 |
| 20 | Acetaminophen, Bis(2,2,6,6-Tetramethyl-4-Piperidyl) Sebacate, Fentanyl, Lidocaine, 4-ANPP | 1 | 0 |
| 21 | Acetaminophen, Fentanyl, Lidocaine, 4-ANPP, NPP, Bis(2,2,6,6-Tetramethyl-4-Piperidyl) Sebacate, Aniline, Mannitol | 1 | 0 |
| 22 | 4-ANPP, Acetaminophen, Fentanyl, Lidocaine | 1 | 0 |
| 23 | 4-ANPP, Acetaminophen, Fentanyl, Lidocaine | 1 | 0 |
| 24 | 4-ANPP, Acetaminophen, Bis(2,2,6,6-Tetramethyl-4-Piperidyl) Sebacate, Fentanyl, Lidocaine, Mannitol, Norfentanyl, Fluorofentanyl | 1 | 0 |
| 25 | Fentanyl, Lidocaine | 1 | 0 |
| 26 | Fentanyl, Lidocaine, 4-ANPP; Aniline; Norfentanyl; Bis(2,2,6,6-Tetramethyl-4-Piperidyl) Sebacate | 1 | 0 |
| 27 | Acetaminophen, Fentanyl, Lidocaine, 4-ANPP | 1 | 0 |
| 28 | 4-ANPP, Acetaminophen, Fentanyl, Lidocaine, Bis(2,2,6,6-Tetramethyl-4-Piperidyl) Sebacate | 1 | 0 |
| 29 | Acetaminophen, Bis(2,2,6,6-Tetramethyl-4-Piperidyl) Sebacate, Fentanyl, Lidocaine, MDMA, Norfentanyl 4-ANPP | 1 | 0 |
| 30 | 4-ANPP, Acetaminophen, Fentanyl, Lidocaine, Mannitol, Norfentanyl | 1 | 0 |
| 31 | Acetaminophen, Bis(2,2,6,6-Tetramethyl-4-Piperidyl) Sebacate, Fentanyl, Lidocaine, MDMA, Norfentanyl, 4-ANPP | 1 | 0 |
| 32 | 4-ANPP, Fentanyl, Lidocaine | 1 | 0 |
| 33 | 4-ANPP, Fentanyl, Lidocaine | 1 | 0 |
| 34 | Fentanyl, Caffeine, 4-ANPP, Mannitol | 0 | 0 |
| 35 | 4-ANPP, Acetaminophen, Fentanyl, Fluorofentanyl | 0 | 0 |
| 36 | 4-ANPP, Acetaminophen, Fentanyl, Fluorofentanyl | 0 | 0 |

**Supplemental Table 2. Drug Contents for Samples with False Positive XTS Results**

For the n=36 samples with a positive XTS results and no xylazine found on DART-MS, the sample contests according to DART-MS are shown. Binary presence/absence indicators are shown for two substances known to cause false positives among XTS in the literature, namely lidocaine and ketamine. Lidocaine was found among n=33, representing 91.7% of false positives. Ketamine was found among n=1 sample.

**
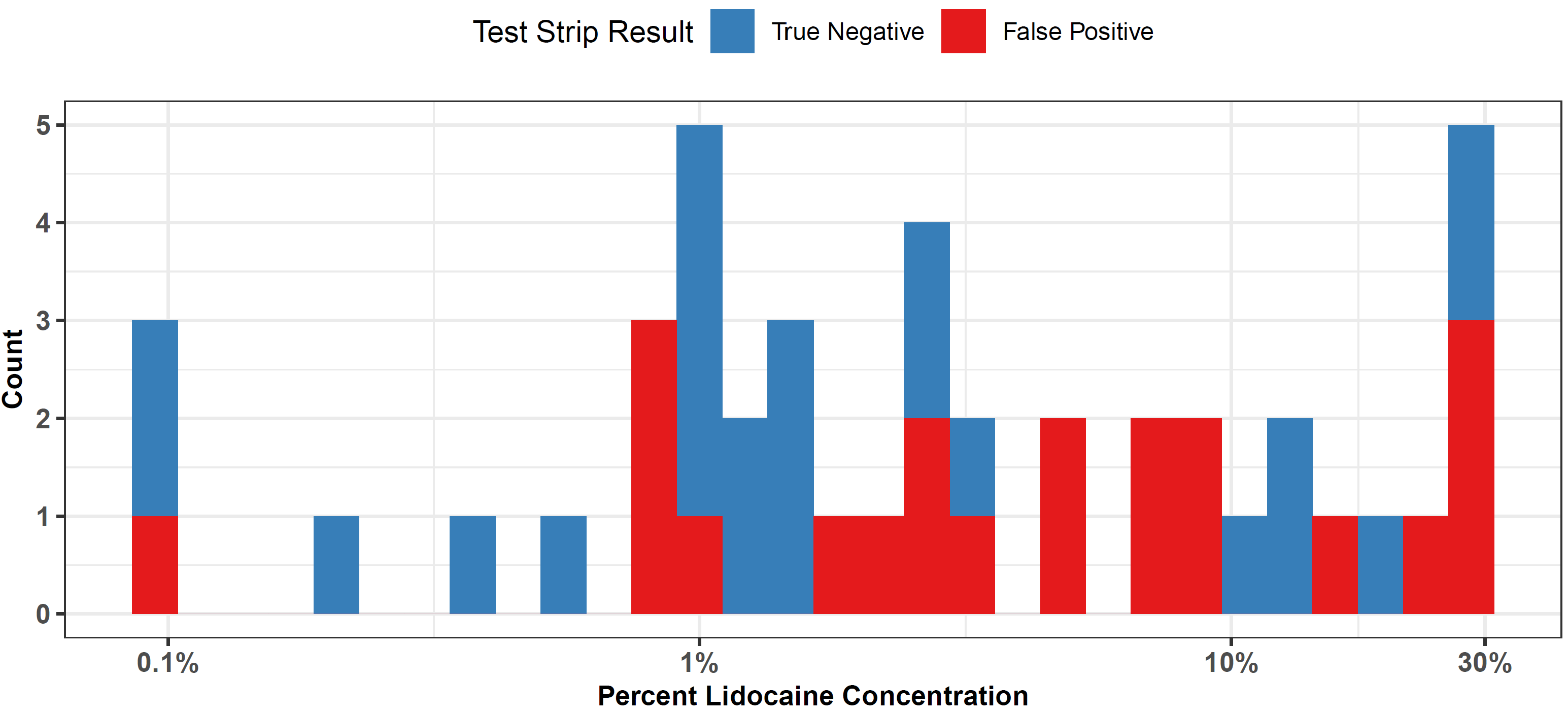
**

**Supplemental Figure 1. Xylazine Test Strip Results by Lidocaine Concentration (Among Xylazine Negative Samples)**

The distribution of lidocaine concentration (by weight) according to LC-MS is shown among samples that 1) did have lidocaine present on DART-MS, and with lidocaine quantified on LC-MS, 2) without xylazine present on DART-MS (meaning all samples should be negative on xylazine test strip), and 3) with xylazine test strip results. A log scale is used on the x-axis to show the percent concentration by mass of lidocaine. Results are shown separate by false positive (shown in red) and true negatives (shown in blue). This figure highlights that false positives, which have been shown in other studies to occur in the presence of lidocaine, occurred at a variety of lidocaine concentrations.

| **XTS** | **DART-MS** | **LC-MS** | **Sample Size** | **Explanation of Circumstances** |
| --- | --- | --- | --- | --- |
| 1 | 1 | 0 | 410 | These samples had xylazine test strips performed in the field, and swabs were sent for DART-MS. LC-MS was not done, either because the participant was unable or unwilling to provide a sufficient mass of sample, or because quantification vials had run out for the day of data collection due to a limited supply when piloting the lab quantification procedures. |
| 0 | 1 | 0 | 384 | These samples had DART-MS testing done. Xylazine test strips were not done, either because participants did not want them to be used, or because they did not believe their sample would contain fentanyl (which would trigger xylazine strip testing). LC-MS was not done, either because the participant was unable or unwilling to provide a sufficient mass of sample, or because quantification vials had run out for the day of data collection due to a limited supply when piloting the lab quantification procedures. |
| 0 | 1 | 1 | 328 | These samples were sent for DART-MS and LC-MS but did not have xylazine test strips performed. This occurred when patients did not wish to engage in test strips, but accepted other testing modalities. |
| 1 | 1 | 1 | 305 | These samples had all testing methodologies used. Sufficient sample was available for LC-MS testing, and participants accepted all 3 modalities. |
| 1 | 0 | 0 | 86 | These samples had xylazine test strips performed, but participants declined confirmatory testing with DART-MS or LC-MS, likely given those modalities do not provide results in real time. |
| 0 | 0 | 0 | 57 | These samples had none of the testing modalities. Instead, they likely obtained only FTIR testing, or only non-xylazine test strips. |
|  |  | **Total** | **1570** |  |

**Supplemental Table 3. Sample Sizes of Combinations of Testing Methodologies Used**

For all samples considered in this study, each combination of testing methodologies is shown as a row, along with the corresponding sample size. An explanation of the circumstances leading to each combination of testing methods is also provided.

**Xylazine Test Strip Result by Xylazine Concentration**

False Negative

or Below Threshold

Test Strip Result

True Positive

**30**

**20**

**10**

**0**

**0.1%**

**1%**

**Percent Xylazine Concentration**

**10%**

**30%**


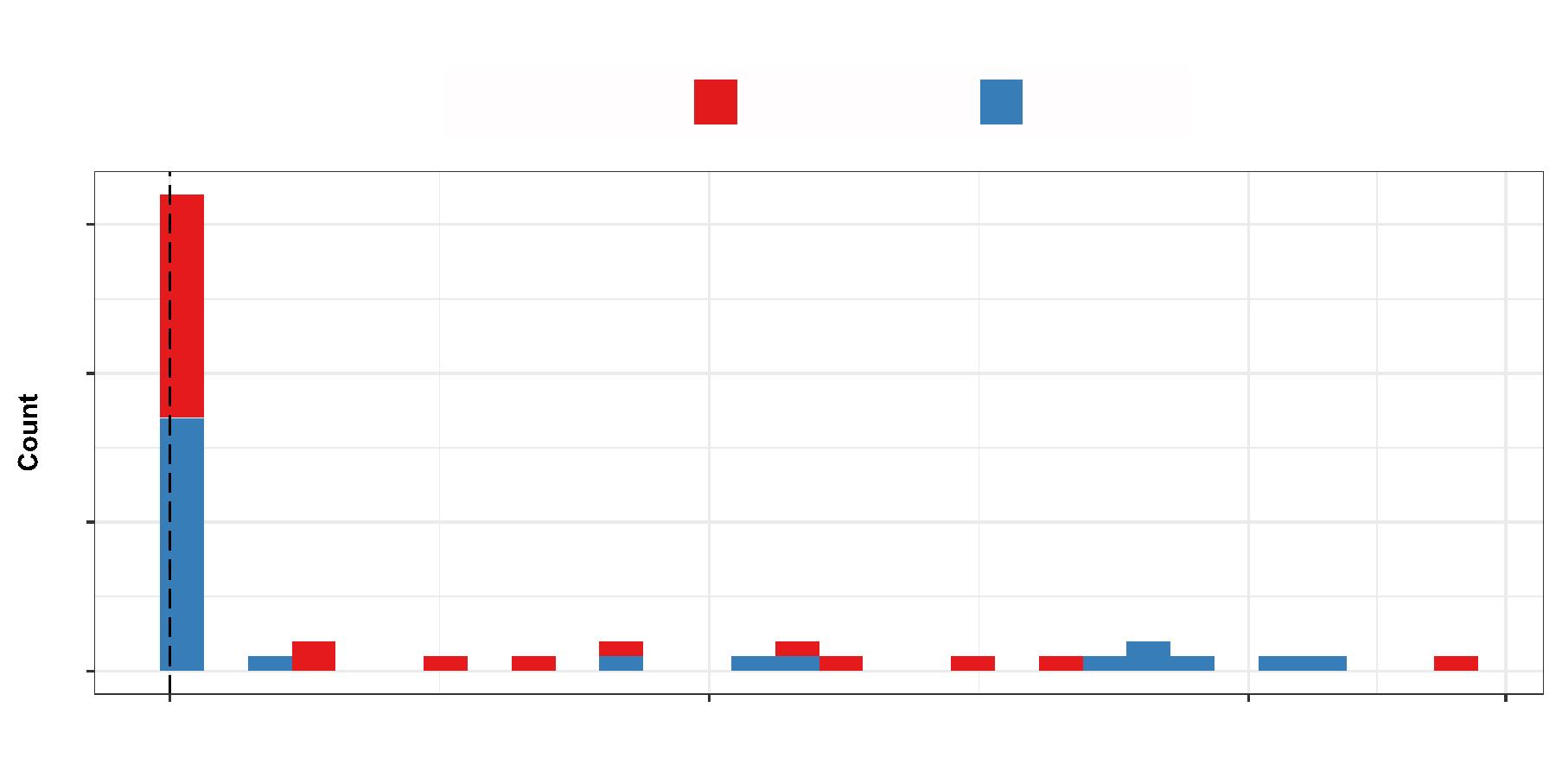

Supplement: Supplementary file 1 — Supplementary Material 1 [file 12954_2026_1396_MOESM1_ESM.docx]
